# 717. Antimicrobial Resistance of Gram-Negative Bacteria in Respiratory Isolates from an Intensive Care Unit in Guatemala

**DOI:** 10.1093/ofid/ofad500.779

**Published:** 2023-11-27

**Authors:** Diego Erdmenger, Luis Rodriguez, Fernando Ochoa, Andres Alfaro, Carolina Portillo, Izabel de Leon, Laura Valenzuela

**Affiliations:** Hospital General San Juan de Dios, Guatemala, Guatemala, Guatemala; Hospital General San Juan de Dios, Guatemala, Guatemala, Guatemala; Hospital General San Juan de Dios, Guatemala, Guatemala, Guatemala; Hospital General San Juan de Dios, Guatemala, Guatemala, Guatemala; Hospital General San de Dios, Guatemala, Jalapa, Guatemala; Hospital General San Juan de Dios, Guatemala, Guatemala, Guatemala; Hospital General San Juan de Dios, Guatemala, Guatemala, Guatemala

## Abstract

**Background:**

Antimicrobial resistance (AMR) among Gram-negative bacteria (GNB) is a growing concern in public health, particularly in ICU settings. Over the past few decades, there has been a significant increase in ESBL and carbapenem resistance in Latin America, which has resulted in higher mortality rates and increased healthcare costs.

One way to understand local bacterial resistance profiles is to examine respiratory isolates, which can serve as a proxy for resistance observed in the clinical setting. It is critical for clinicians to be aware of these profiles in order to make informed decisions about treatment options.

**Methods:**

A retrospective study was conducted on all respiratory isolates from the ICU of a third-level national hospital over a 6-month period. All respiratory isolates were analyzed for to identify microorganisms and their AMR to selected antibiotics, including Ceftriaxone (CRO), Ceftazidime (CAZ), Cefepime (CEF) and carbapenems.

**Results:**

A total of 500 isolates were obtained during the study period, resulting in the isolation of 988 microorganisms. GNB represented 92.3% (n=912) of these isolates. *Pseudomonas* spp was the most common GNB identified (28.07%), followed by *Acinetobacter* (26.97%) and *Klebsiella spp* (20.94%).

*Stenotrophomona* isolates were excluded from the analysis as resistance profiling for this organism is not routinely performed in the institution, leaving a total of 868 isolates for AMR analysis.

A total of 527 GNB isolates (60.78%) were identified as resistant to both 3^rd^ generation cephalosporines and CEF. Strains with this resistance pattern were considered highly suggestive of ESBL production. Moreover, 66.78% (n=579) of GNB’s analyzed evidenced resistance to carbapenems, with 13.61% (n=118) of strains having been identified as carbapenemase-producing bacteria.

Some GNB exhibited alarming levels of resistance as evidenced in table 1.

**Table 1.**
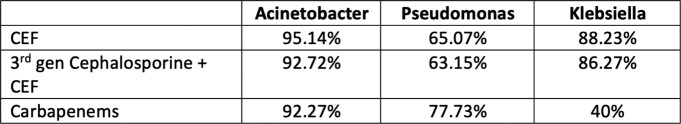
Resistance of selected GNB Percentage of bacterial strains from respiratory isolates resistant to selected antibiotics.

**Conclusion:**

The high level or resistance observed in GNB from respiratory isolates in this study is concordant with other data from Latin America. While the prevalence of GNB in respiratory isolates does not necessarily correlates with clinical infection rates, the high level of resistance found may be an indicator of the AMR epidemiology of infections in this ICU.

**Disclosures:**

**All Authors**: No reported disclosures

